# Supplementary material for: An intranasal nanoparticle STING agonist protects against respiratory viruses in animal models
Source: Nat Commun. 2024 Jul 18;15:6053. doi: 10.1038/s41467-024-50234-y (PMC11258242; doi:10.1038/s41467-024-50234-y)
Supplement: Supplementary file 1 — Supplementary Information [file 41467_2024_50234_MOESM1_ESM.pdf]

SUPPLEMENTARY INFORMATION

An intranasal STING agonist nanoparticle protects against respiratory viruses in animal models

Ankita Leekha[1], Arash Saeedi[1], Monish Kumar[1], KM Samiur Rahman Sefat[1], Melisa Martinez-Paniagua[1], Hui Meng[2], Mohsen Fathi[1], Rohan Kulkarni[1], Kate Reichel[1], Sujit Biswas[3], Daphne Tsitoura[4], Xinli Liu[3], Laurence J.N. Cooper[4], Courtney M. Sands[5], Vallabh E. Das[2], Manu Sebastian[4], Brett L. Hurst[6], and Navin Varadarajan[1†]

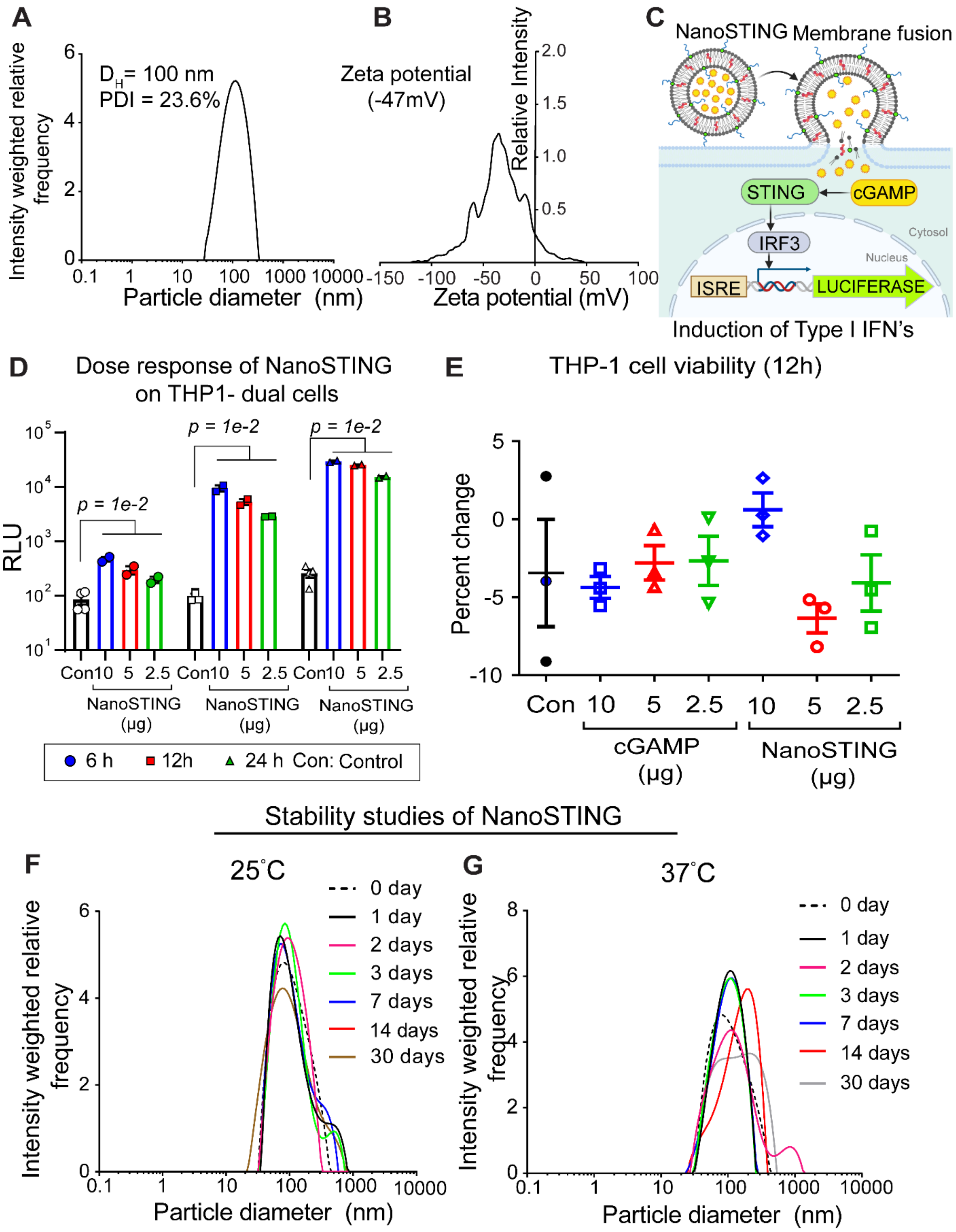
A
Intensity weighted relative frequency
D_H = 100 nm
PDI = 23.6%
Particle diameter (nm)
B
Zeta potential
(-47mV)
Relative Intensity
Zeta potential (mV)
C
NanoSTING
Membrane fusion
STING
cGAMP
IRF3
Cytosol
Nucleus
ISRE
LUCIFERASE
Induction of Type I IFN's
D
Dose response of NanoSTING on THP1- dual cells
p = 1e-2
RLU
Con 10 5 2.5
NanoSTING (µg)
6 h
12h
24 h
Con: Control
E
THP-1 cell viability (12h)
Percent change
Con 10 5 2.5 10 5 2.5
cGAMP (µg)
NanoSTING (µg)
Stability studies of NanoSTING
F
25˚C
0 day
1 day
2 days
3 days
7 days
14 days
30 days
Intensity weighted relative frequency
Particle diameter (nm)
G
37˚C
0 day
1 day
2 days
3 days
7 days
14 days
30 days
Intensity weighted relative frequency
Particle diameter (nm)

Supplementary Fig. 1: Characterization of NanoSTING (related to Fig. 1)

(**A**) Size distribution of NanoSTING measured by DLS. (**B**) The zeta potential of the NanoSTING was measured by electrophoretic light scattering (ELS). (**C**) Mechanisms of NanoSTING delivery and signaling pathways. THP1-dual cells stably express the secreted form of luciferase under the Control of a synthetic interferon-responsive promoter. Activation of the IRF pathway leads to the secretion of luciferase in the cell culture supernatant. (**D**) Kinetics of the induction of luciferase in THP1-dual cells by varying concentrations of the NanoSTING. RLU: relative light units. (**E**) Cell viability of cGAMP and NanoSTING on THP1-dual cells. (**F, G**) Distribution of NanoSTING liposomal particle sizes at 25°C and 37°C measured by dynamic light scattering (DLS). Analysis was performed using a two-tailed Mann-Whitney U-test. Individual data points represent technical replicates; vertical bars show mean values with error bars representing SEM. Mann-Whitney U-test: ****p < 0.0001; ***p < 0.001; **p < 0.01; *p < 0.05; ns: not significant. Gender was not tested as a variable, and only female mice were included in the study. See also Supplementary Table 1. Color codes: 10μg NanoSTING (blue), 5μg NanoSTING (red), 2.5μg NanoSTING (blue) and Control (black). Supp. Fig. 1B-Created with BioRender.com released under a Creative Commons Attribution-NonCommercial-NoDerivs 4.0 International license (https://creativecommons.org/licenses/by-nc-nd/4.0/deed.en)

Source data are provided as a Source Data file.

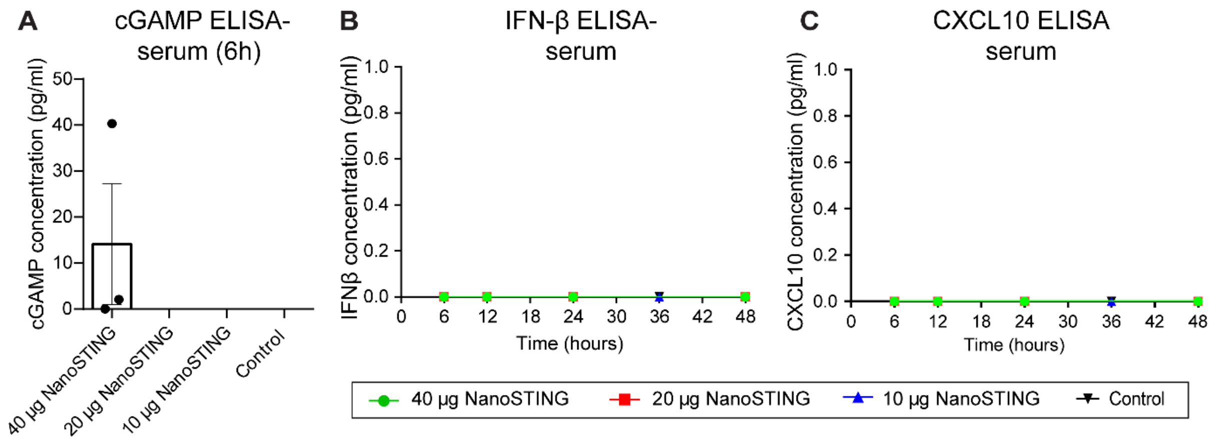

Supplementary Fig. 2: ELISA results confirmed that stimulation of the innate immunity was localized and not systemic (related to Fig. 1)

(**A**) Quantification of cGAMP in the mouse serum (*BALB/c)* after treatment with NanoSTING. (**B**) Detection of IFN-β concentration in the mouse serum using quantitative ELISA. (**C**) Detection of CXCL10 levels in the mouse serum using quantitative ELISA. The analysis was performed using a two-tailed Mann-Whitney U-test. Vertical bars show mean values with error bar representing SEM. Mann-Whitney U-test: ****p < 0.0001; ***p < 0.001; **p < 0.01; *p < 0.05; ns, not significant. Individual data points represent independent biological replicates taken from discrete samples. Data presented as combined results from one (A-C) independent animal experiment. Gender was not tested as a variable, and only female mice were included in the study. Color codes: 40µg NanoSTING (green), 20µg NanoSTING (red), 10µg NanoSTING (blue) and Control (black).

Number of animals used: n=3-8/group

Source data are provided as a Source Data file.

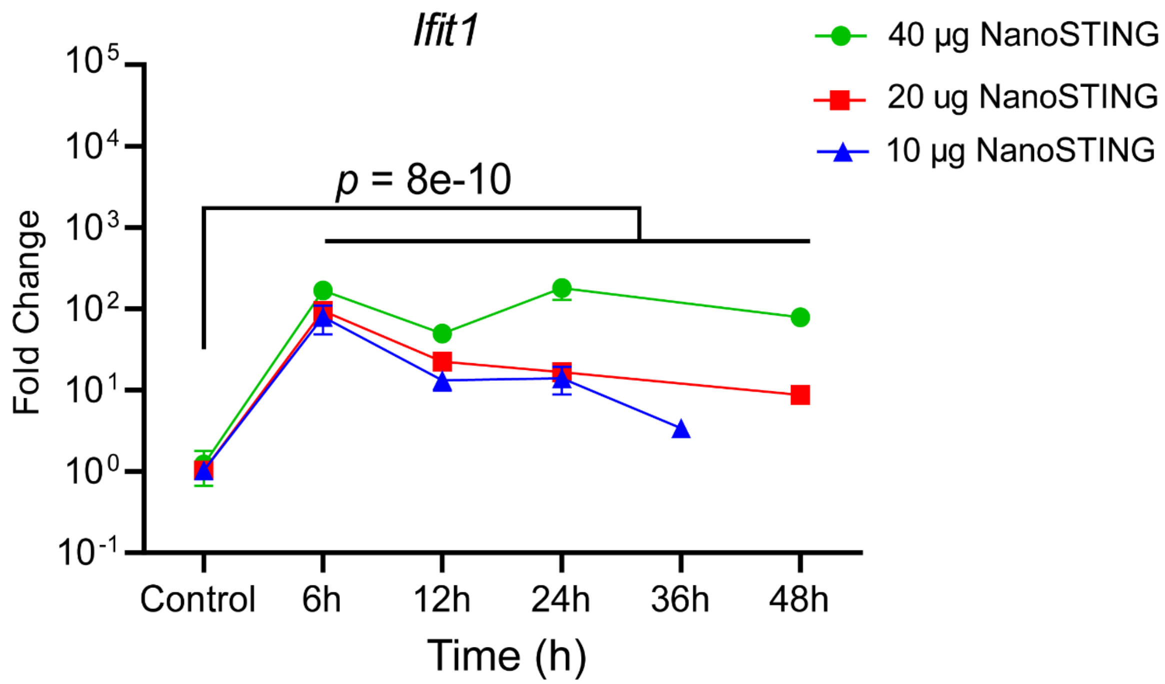

Supplementary Fig. 3: Real-time qRT-PCR for fold induction of *Ifit1* mRNA from NanoSTING treated mice compared with control mice nasal turbinates (related to Fig. 1)

Analysis was performed using a two-tailed Mann-Whitney U-test for fold changes in gene expression. Mann-Whitney U-test: ****p < 0.0001; ***p < 0.001; **p < 0.01; *p < 0.05; ns: not significant. Individual data points represent independent biological replicates taken from discrete samples. Color codes: 40µg NanoSTING (green), 20µg NanoSTING (red), 10 µg NanoSTING (Blue). Data presented as combined results from one independent animal experiment. Gender was not tested as a variable, and only female mice were included in the study. Color codes: 40µg NanoSTING (green), 20µg NanoSTING (red), 10µg NanoSTING (blue) and Control (black).

Number of animals used: n=3-4/group

Source data are provided as a Source Data file.

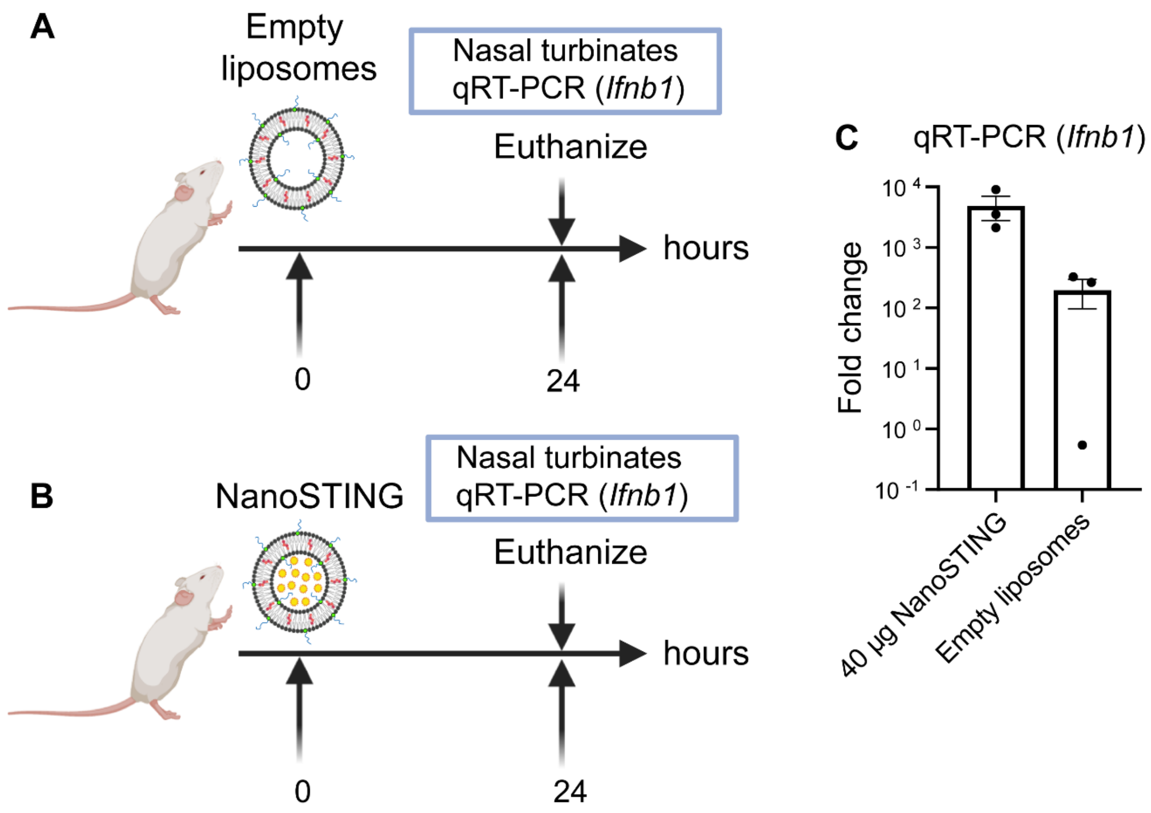

Supplementary Fig. 4: cGAMP is essential for robust induction of *Ifnb1* in the nasal turbinates

(**A, B**) *BALB/c* mice (n=3 in each group) were intranasally administered with empty liposomes or 40 µg of NanoSTING followed by euthanasia post-24 h. We collected nasal turbinates for RT-qPCR (*Ifnb1).* (**C**) Fold change in gene expression for mice intranasally administered with 40 µg NanoSTING or empty liposomes was quantified using RNA extracted from nasal turbinates by qRT-PCR (Primer sequences are provided in Supplementary Table 2). Individual data points represent independent biological replicates taken from discrete samples. Data presented as combined results from one (B) independent animal experiment. Analysis was performed using a two-tailed Mann-Whitney U-test. Mann-Whitney U-test: ****p < 0.0001; ***p < 0.001; **p < 0.01; *p < 0.05; ns: not significant. Gender was not tested as a variable, and only female mice were included in the study. Individual data points represent independent biological replicates taken from separate animals. Supp. Fig. 4A-Created with BioRender.com released under a Creative Commons Attribution-NonCommercial-NoDerivs 4.0 International license (https://creativecommons.org/licenses/by-nc-nd/4.0/deed.en)

Number of animals used: n=3/group

Source data are provided as a Source Data file.

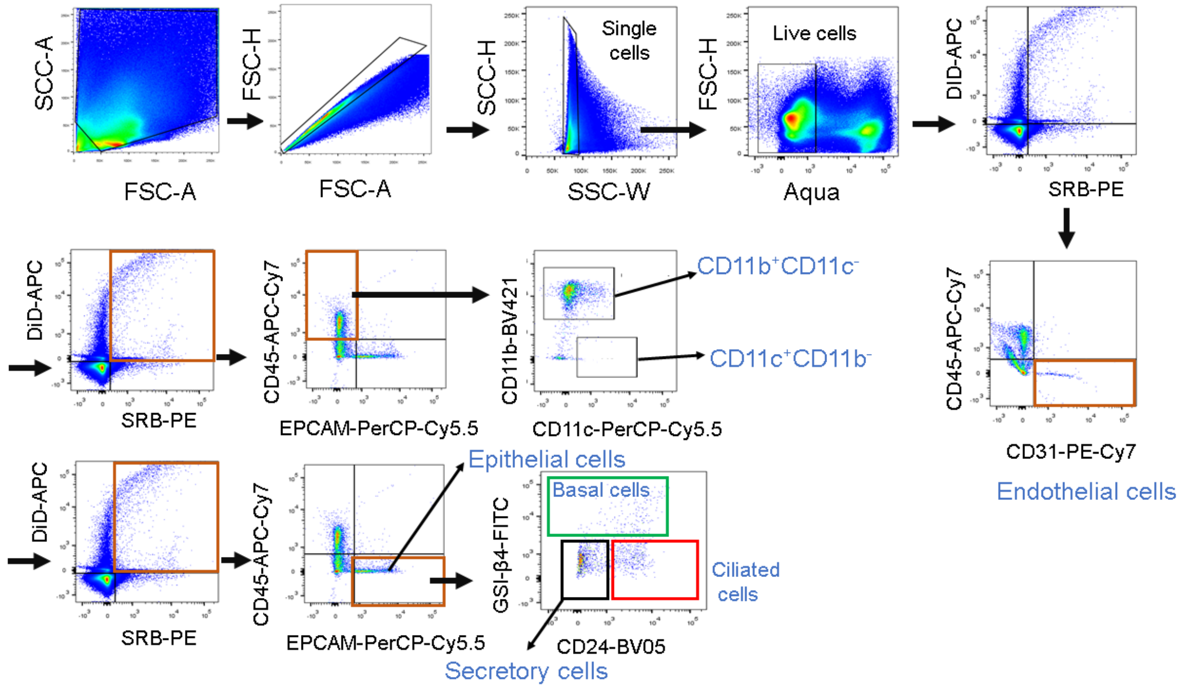

Supplementary Fig. 5: Gating strategy for the identification of endothelial cells, macrophages, and the different subsets of epithelial cells by flow-cytometry (related to Fig. 2).

As shown in Supplementary Figure 5, FSC-A vs FSC-H and SSC-H vs SSC-W parameters were used to exclude doublets. In addition, the live cell population was selected as Live/Dead Aqua negative cells. Subsequently, we gated on $DiD^{+}SRB^{+}$ lung and nasal tissue cells. In both tissues, we specifically focused on four major subtypes of cells: epithelial cells ($CD45^{-}EPCAM^{+}CD31^{-}$), endothelial cells ($CD45^{-}CD31^{+}$), and two myeloid subsets: $CD45^{+}EPCAM^{-}CD11b^{+}CD11c^{-}$ cells and $CD45^{+}EPCAM^{-}CD11c^{+}CD11b^{-}$ cells. We investigated the cell types of the murine tracheal epithelium (basal cells, secretory cells & ciliated cells). SSC-A: side scatter area, FSC-A: forward scatter area, FSC-H: forward scatter height, SSC-H: side scatter height, SSC-W: side scatter width.

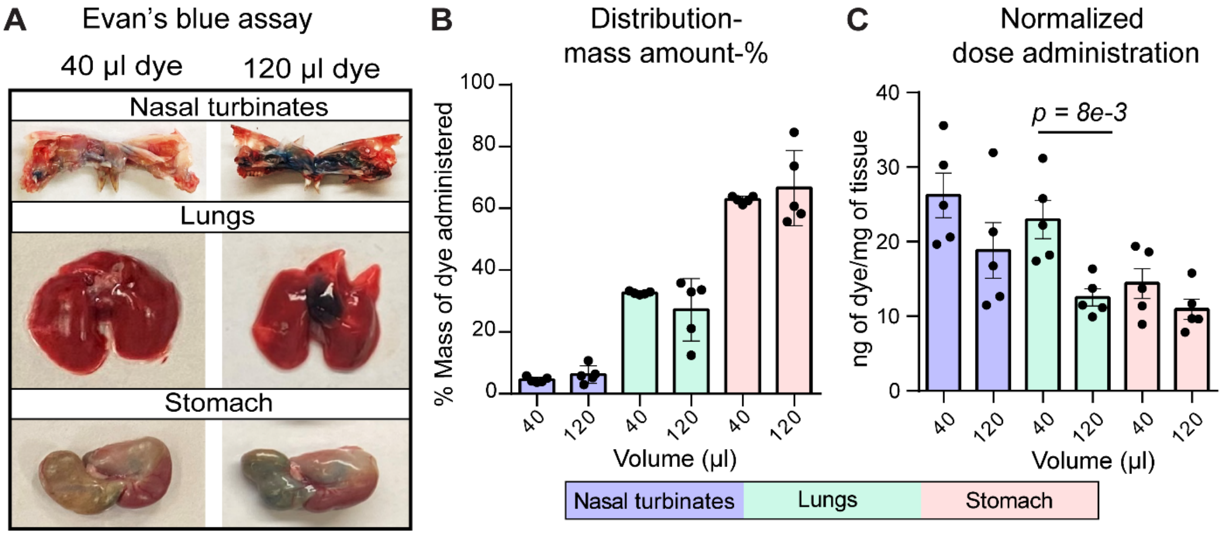

Supplementary Fig. 6: Distribution studies for NanoSTING (related to Fig. 4)

(**A**) Evan's blue dye assay. Hamsters (n=5/group) were intranasally administered with 0.125% Evans blue dye in PBS (40 µL and 120 µL). Representative images of nasal turbinates, lungs, and stomach dissected 2 min later are shown. (**B, C**) We treated the supernatants from homogenized lungs and stomach with trichloroacetic acid and analyzed absorbance at 620 nm. We interpolated the concentrations of dye from a standard curve. For (**B**) & (**C**), the analysis was performed using a two-tailed Mann-Whitney U-test. Individual data points represent independent biological replicates taken from discrete samples; vertical bars show mean values with error bars representing SEM. Mann-Whitney U-test: ****p < 0.0001; ***p < 0.001; **p < 0.01; *p < 0.05; ns: not significant. Individual data points represent independent biological replicates taken from discrete samples. Data has been normalized using control samples from untreated mice. Data presented as combined results from one (B-C) independent animal experiment. Three male and two female hamsters were taken for the study. Color codes: Nasal turbinates (purple), Lungs (sea-green), stomach (pink) and Control (black).

Number of animals used: n=5/group

Source data are provided as a Source Data file.

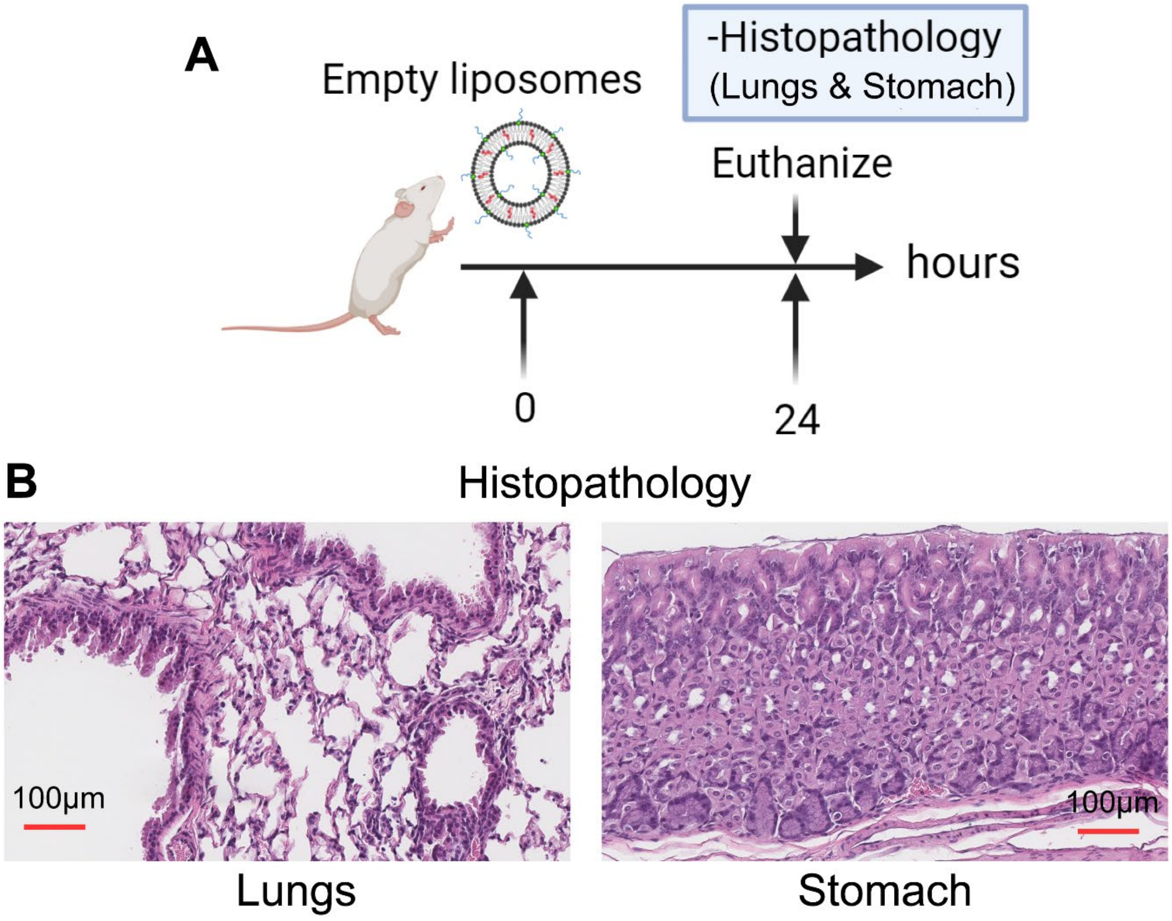

Supplementary Fig. 7: Assessment of the safety of liposomes in mice

(A) Groups of three *BALB/c* mice were intranasally administered liposomes (without encapsulated cGAMP), and animals were euthanized post 24h. Lungs and stomach were collected for histopathological analysis. **(B)** Representative H & E images of the lung and stomach of mice; all images were acquired at 20x; scale bar, 100 µm. Gender was not tested as a variable; only female mice were included in the study. Supp. Fig. 7A-Created with BioRender.com released under a Creative Commons Attribution-NonCommercial-NoDerivs 4.0 International license (https://creativecommons.org/licenses/by-nc-nd/4.0/deed.en)

Number of animals used: n=3/group

Source data are provided as a Source Data file.

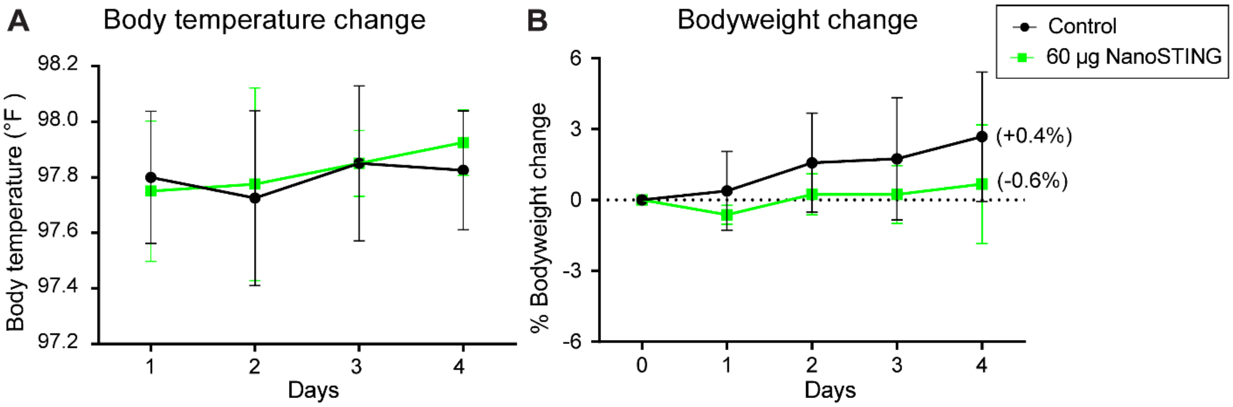

Supplementary Fig. 8: Safety studies for NanoSTING (related to Fig. 4)

(A, B) We administered groups of animals with daily doses of 60 µg of NanoSTING intranasally (n=4/group) or PBS (n=4/group) for four consecutive days. We monitored the hamsters daily for body weight and body temperature changes. We euthanized the hamsters on Day 5 after administering the last dose on Day 4, followed by the collection of lungs. Bodyweight change, body temperature change, qRT-PCR for lungs (Supplementary Fig. 10), and mRNA sequencing (Fig. 3) were primary endpoints. Individual data points represent independent biological replicates taken from discrete samples; vertical bars show mean values with error bars representing SEM. Data presented as combined results from one (A-B) independent animal experiment. Number of animals used: n=4/group**.** For **A** & **B**, data was compared via mixed-effects model for repeated measures analysis. Lines depict group mean body weight temperature and weight change from day 0; error bars represent SEM. Gender was tested as a variable, and an equal number of male and female hamsters were included in the study. Color codes: 60µg NanoSTING (Green), Control (black).

Number of animals used: n=5/group

Source data are provided as a Source Data file.

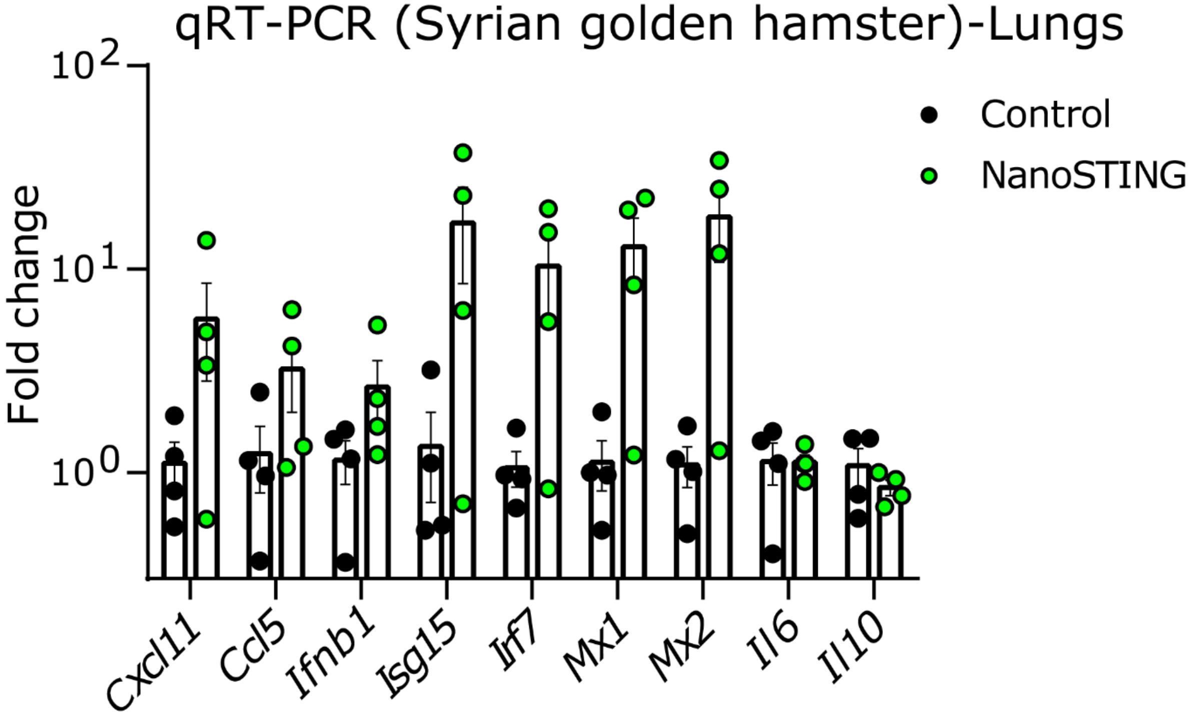

Supplementary Fig. 9: Upregulation of interferon-stimulated genes (*Cxcl11, Ccl5, Ifnb1, Isg15, Irf7, Mx1, Mx2, Il6,* and *Il10)* in the lungs of hamsters upon intranasal NanoSTING treatment (related to Fig. 4)

Analysis was performed using a two-tailed Mann-Whitney U-test for fold changes in gene expression. Individual data points represent independent biological replicates taken from discrete samples; vertical bars show mean values with error bars representing SEM. Each dot represents an individual hamster. ****p < 0.0001; ***p < 0.001; **p < 0.01; *p < 0.05; ns, not significant. Data presented as combined results from one independent animal experiment. Color codes: Control (Black data points), NanoSTING (Green data points). See Supplementary Table 3 for a list of primers used. Gender was tested as a variable, and an equal number of male and female hamsters were included in the study.

Number of animals used: n=4/group.

Source data are provided as a Source Data file.

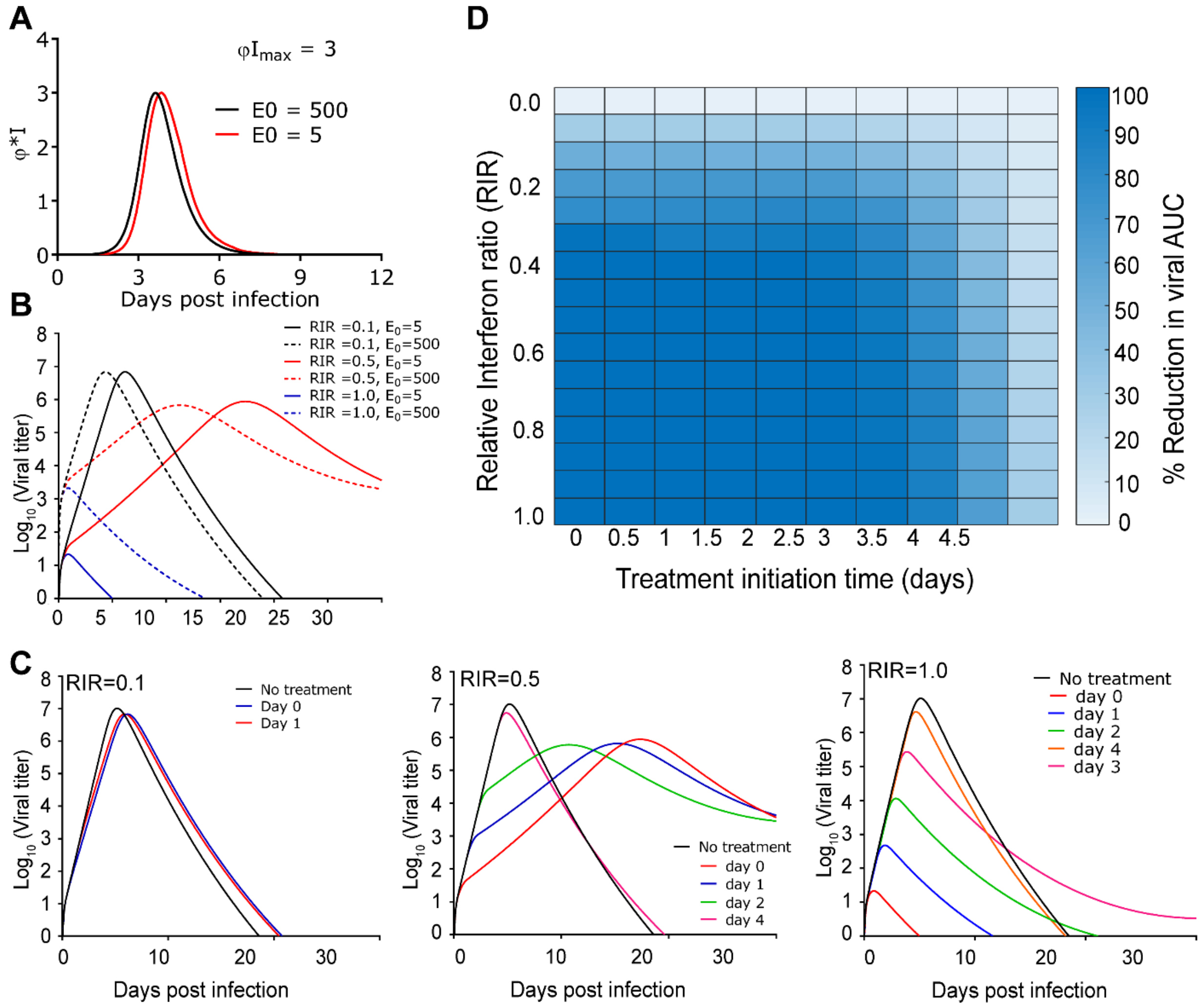

Supplementary Fig. 10: Quantitative modeling predicts reduction in viral titers upon administration of NanoSTING (related to Fig. 5)

(**A**) Peak natural response is independent of the initial viral titer. (**B**) Viral dynamics are independent of initial viral titer upon treatment with NanoSTING. E0 is the initial number of infected cells upon viral infection, which is a surrogate for viral titer. (**C**) Evolution of viral dynamics with varying NanoSTING efficacies and time of treatment. (**D**) Heatmap of viral AUC with varying NanoSTING efficacy and treatment initiation time when NanoSTING effects last 48 h after treatment initiation.

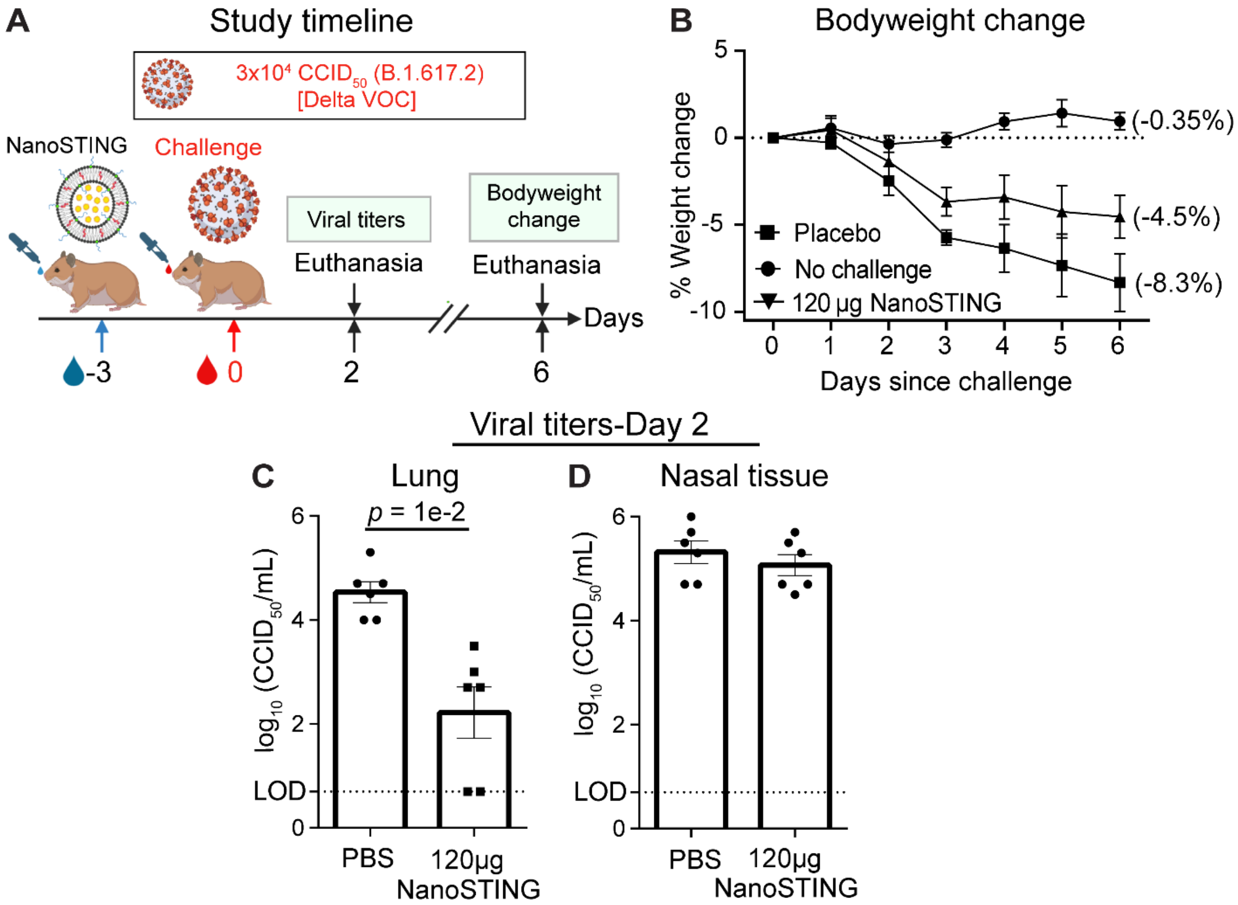

Supplementary Fig. 11: Pre-treatment of NanoSTING protects against challenge with SARS-CoV-2 delta VOC (B.1.617.2) (related to Fig. 6)

(**A**) Experimental setup. We treated groups of 12 hamsters, each with a single dose of NanoSTING (120 µg) and 72 h later challenged with ~3 x 10^4 $CCID_{50}$ of SARS-CoV-2 virus (Delta VOC-B.1.617.2). We monitored animal weight changes daily for 6 days. Bodyweight changes and viral titers in the lungs and nasal tissue were primary endpoints. (**B**) The percentage of bodyweight change in NanoSTING-treated animals compared to the control. (**C, D**) Viral titers were quantified in the lung and nasal tissue by end point titration assay post day 2 after the challenge. The dotted line indicates the limit of detection of the assay (LOD). For (C) & (D), analysis was performed using a two-tailed Mann-Whitney U-test. Individual data points represent independent biological replicates taken from discrete samples; vertical bars show mean values with error bars representing SEM. Each dot represents an individual hamster. For (B), weight data was compared via a mixed-effects model for repeated measures analysis. Lines depict group mean body weight change from day 0; error bars represent SEM. Asterisks indicate significance compared to

the placebo-treated animals at each time point. ****$p < 0.0001$; ***$p < 0.001$; **$p < 0.01$; *$p < 0.05$; ns, not significant. Data presented as combined results from one (B-D) independent animal experiment. Gender was tested as a variable with an equal number of male and female hamsters included in the study. Supp. Fig. 11A-Created with BioRender.com released under a Creative Commons Attribution-NonCommercial-NoDerivs 4.0 International license (https://creativecommons.org/licenses/by-nc-nd/4.0/deed.en)

Number of animals used: n=12/group.

Source data are provided as a Source Data file.

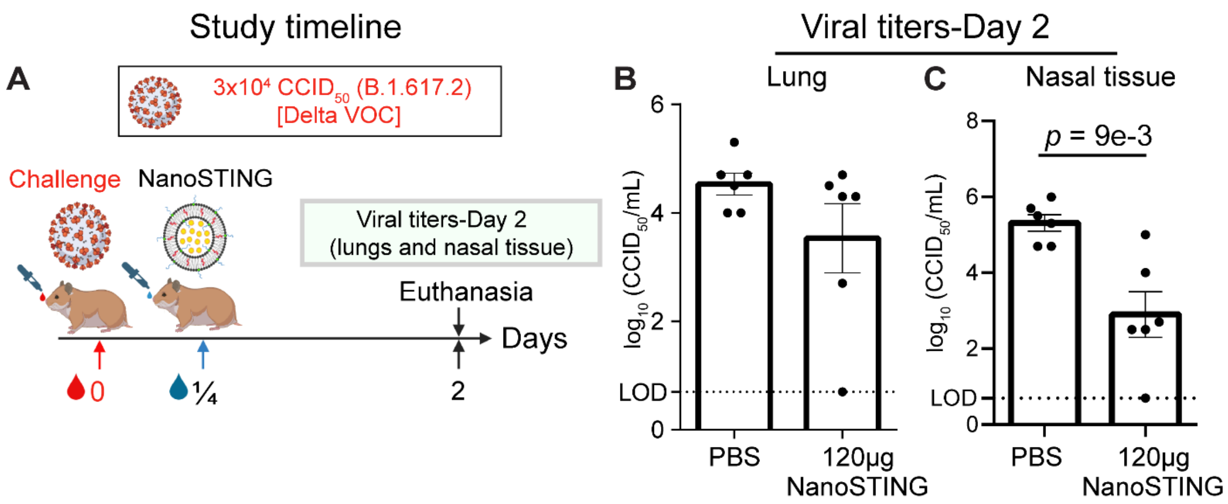

Supplementary Fig. 12: Treatment with NanoSTING protects against challenge with SARS-CoV2 Delta VOC (B.1.617.2) (related to Fig. 6)

(**A**) Experimental setup. We challenged groups of 12 hamsters, each with ~3 x 10^4 SARS-CoV-2 (Delta VOC-B.1.617) and 6 h later treated with a single dose of NanoSTING (120 µg). We euthanized the animals post day 2 of infection and determined viral titers in the lungs and nasal tissue. (**B, C**) Viral titers were quantified in the lung and nasal tissue by plaque assay post day 2 of infection. The dotted line indicates the limit of detection of the assay (LOD). For (B) & (C), analysis was performed using a two-tailed Mann-Whitney U-test. Individual data points represent independent biological replicates taken from discrete samples; vertical bars show mean values with error bars representing SEM. Each dot represents an individual hamster. Asterisks indicate significance compared to the placebo-treated animals. ****p < 0.0001; ***p < 0.001; **p < 0.01; *p < 0.05; ns: not significant. Data presented as combined results from one (B-D) independent animal experiment. Gender was tested as a variable, and an equal number of male and female hamsters included in the study. Supp. Fig. 12A-Created with BioRender.com released under a Creative Commons Attribution-NonCommercial-NoDerivs 4.0 International license (https://creativecommons.org/licenses/by-nc-nd/4.0/deed.en)

Number of animals used: n=12/group.

Source data are provided as a Source Data file.

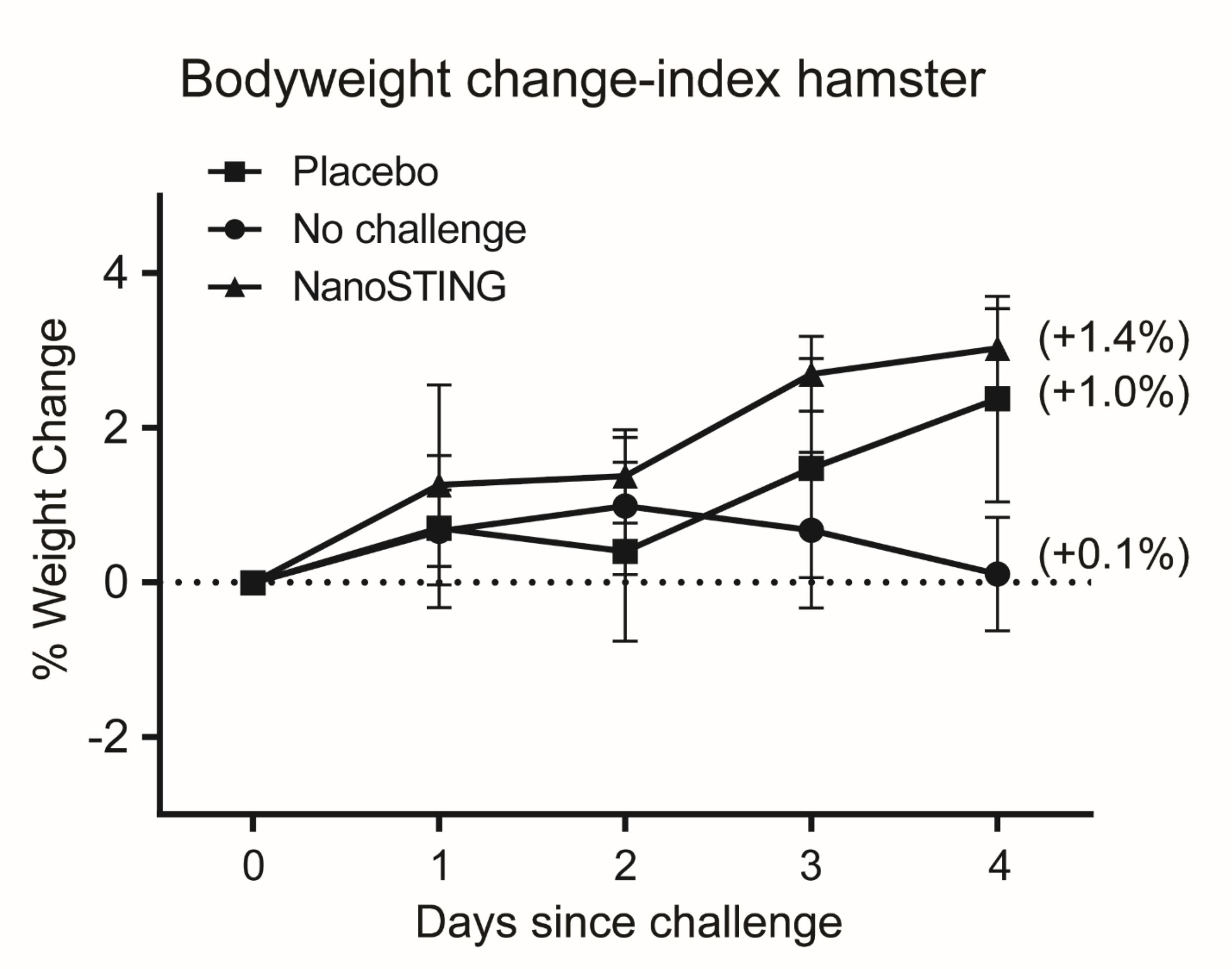

Supplementary Fig.13: Longitudinal measurements of the bodyweight of index hamsters intranasally infected with SARS-CoV-2 Omicron VOC (B.1.1.529) (related to Fig. 8)

Weight data was compared via mixed-effects model for repeated measures analysis. Lines depict group mean body weight change from day 0; error bars represent SEM. Asterisks indicate significance compared to the placebo-treated animals at each time point. ****$p < 0.0001$; ***$p < 0.001$; **$p < 0.01$; *$p < 0.05$; ns: not significant. Gender was tested as a variable, and an equal number of male and female hamsters were included in the study.

Number of animals used: n=8/group.

Source data are provided as a Source Data file.

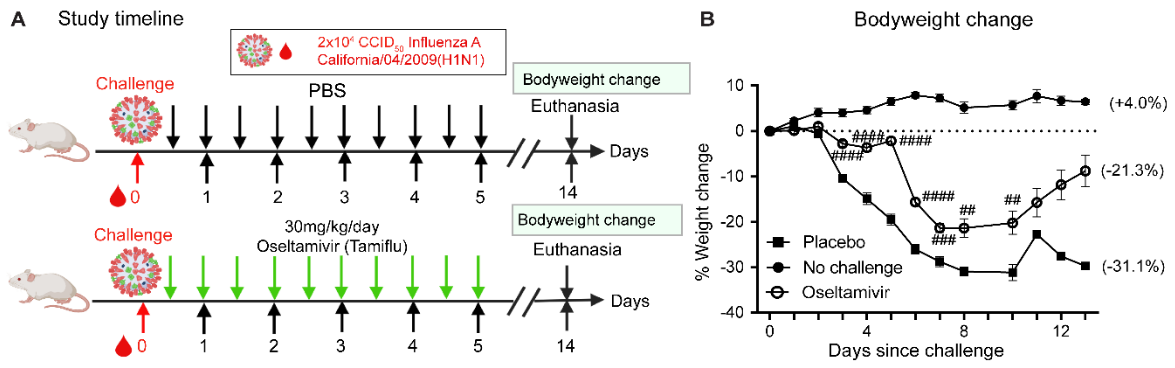

Supplementary Fig. 14: Treatment with Oseltamivir offers moderate protection against Influenza A (related to Fig. 9)

Groups of ten *BALB/c* mice were challenged intranasally with ~2 x 10^4 of Influenza A/California/04/2009 (H1N1dpm) virus and treated with oseltamivir, twice daily for five days. (**B**) Percent weight change of the different groups of mice. Weight data was compared via mixed-effects model for repeated measures analysis. Lines depict group mean body weight change from day 0; error bars represent SEM. Pound sign show statistical significant differences between the Oseltamivir-treated group and placebo-treated animals. ****p < 0.0001; ***p < 0.001; **p < 0.01; *p < 0.05; ns: not significant. Pound sign show statistical significant differences between Oseltamivir-treated group and placebo-treated animals. For B, the exact p-values comparing the Oseltamivir group to the Placebo group are as follows: Day 3: p = 6e-7, Day 4: p=3e-6, Day 5: p = 4e-9, Day 6: p = 1e-6, Day 7: p = 2e-4, Day 8: p = 2e-3 and Day 10: p = 7e-3. Data presented as combined results from one independent animal experiment. Gender was tested as a variable with an equal number of male and female mice included in the study. Supp. Fig. 14A-Created with BioRender.com released under a Creative Commons Attribution-NonCommercial-NoDerivs 4.0 International license (https://creativecommons.org/licenses/by-nc-nd/4.0/deed.en)

Number of animals used: n=10/group.

Source data are provided as a Source Data file.

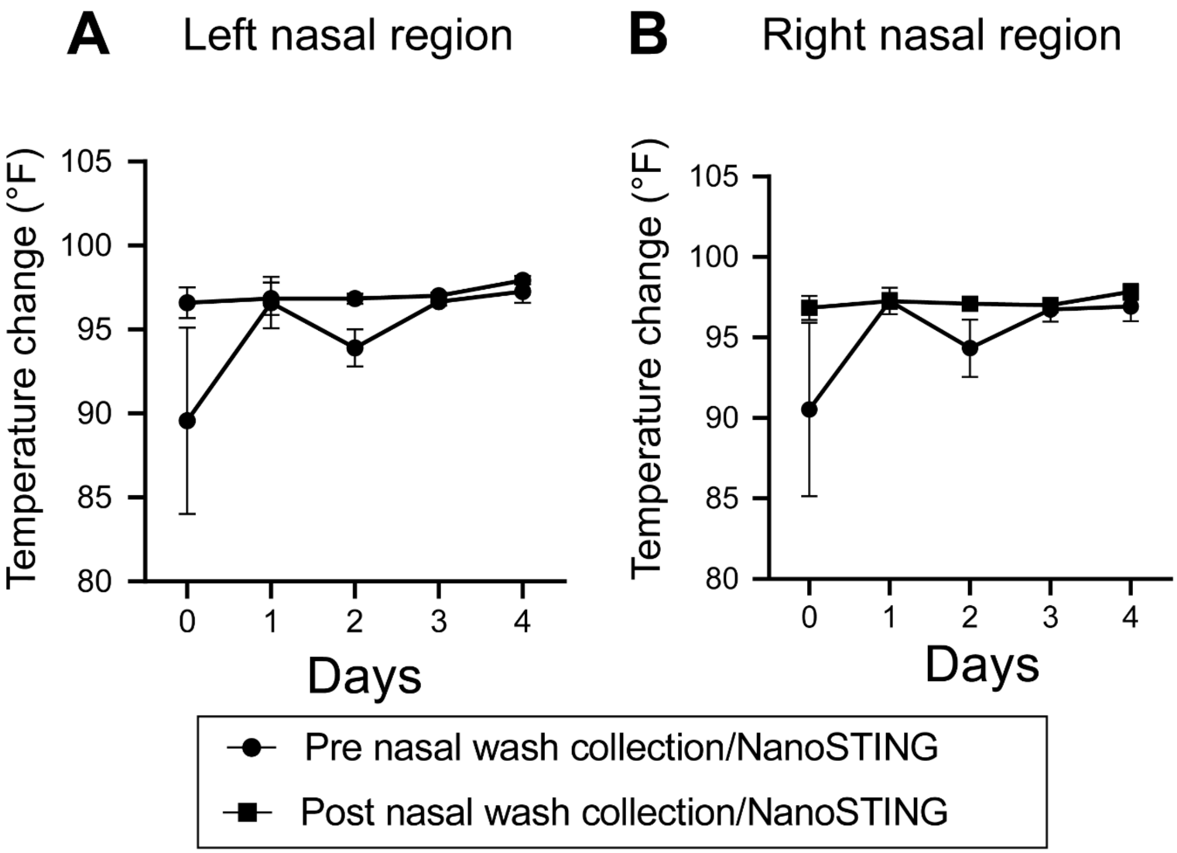

Supplementary Fig. 15: Nasal area temperature monitoring pre and post-nasal wash collection/NanoSTING treatment in rhesus macaques (RMs) (related to Fig. 10)

Experimental set up: We administered one group (n=4/group) of Rhesus macaques (RM's) with two doses of NanoSTING (0.1 mg/kg-range: 0.06-0.14mg/kg) administered intranasally on day 0 and day 2, and we monitored the animals until day 4 for changes in body weight, body temperature and nasal area temperature. (**A**) Monitoring of left nasal area temperature pre and post-nasal wash collection/NanoSTING treatment. (**B**) Monitoring of right nasal area temperature pre and post-nasal wash collection/NanoSTING treatment. Data presented as combined results from one independent animal experiment. Three males and one female NHP were included in this study.

Number of animals used: n=4/group

Source data are provided as a Source Data file.

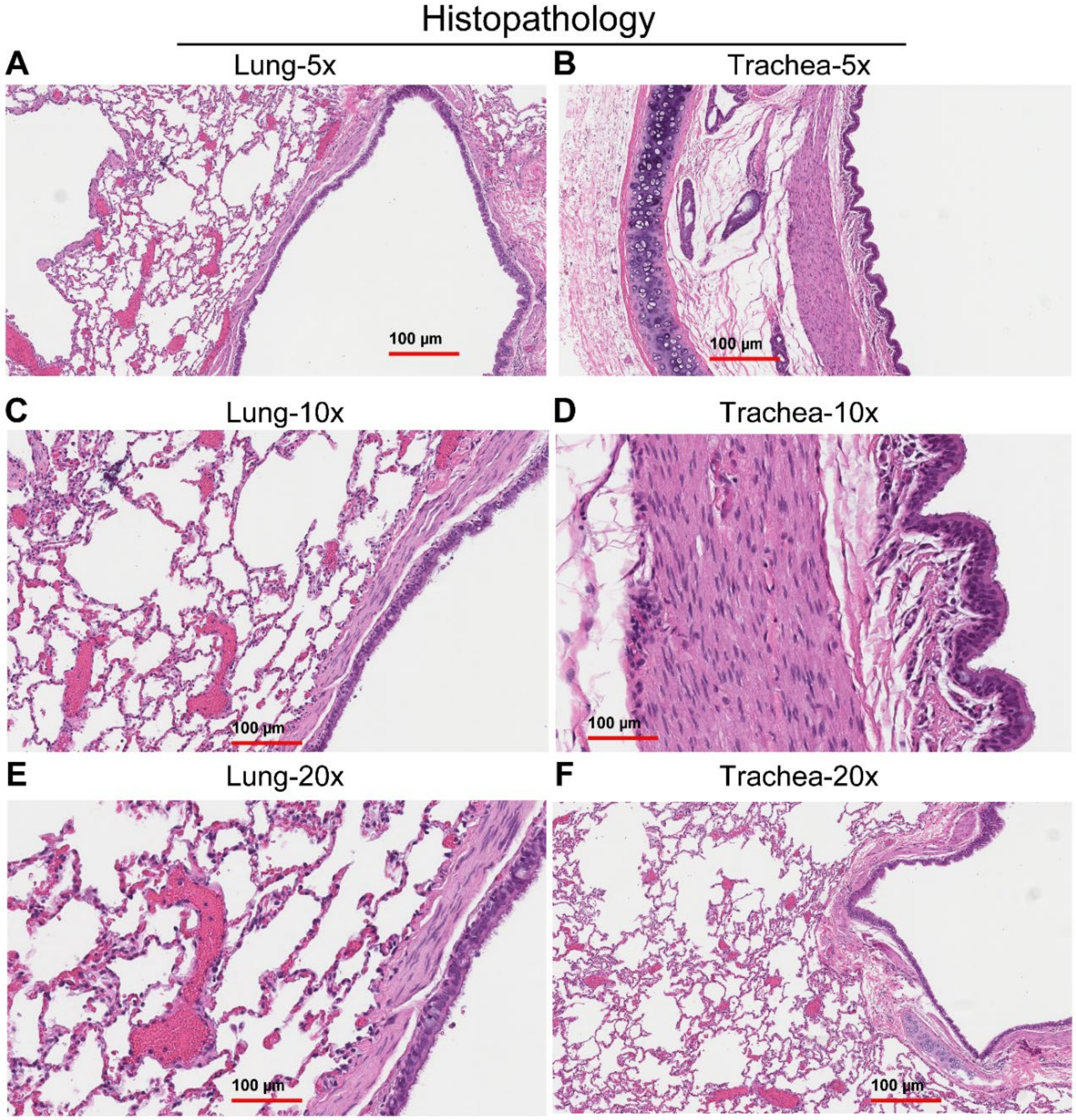

Supplementary Fig. 16: Representative H&E images of the lungs and trachea of RM's treated with two doses of NanoSTING (related to Fig. 10)

Experimental set up: We administered one group (n=4/group) of Rhesus macaques (RM's) with two doses of NanoSTING (0.1 mg/kg-range: 0.06-0.14mg/kg) administered intranasally on day 0 and day 2 and we monitored the animals until day 4 and euthanized one of the animals on day 4 to assess the histopathological changes in the lungs and trachea. (**A, B**) Representative H & E images of the lung and trachea acquired at A & B-5x; (**C & D**) Representative hematoxylin and eosin H & E images of the lung and trachea acquired at 10x (**E & F**) Representative H & E images of the lung and trachea acquired at

20x. Scale bar-100 μm. Data presented as combined results from one independent animal experiment. Three males and one female NHP were included in this study.

Number of animals used: n=4/group

Source data are provided as a Source Data file.

Day	Temperature (25 °C)			Temperature (37 °C)		
	Particle size (DH)	PDI (%)	Zeta potential (mV)	Particle size (DH)	PDI (%)	Zeta potential (mV)
**0**	98	25.1	-45	98	25.1	-45
**1**	108	25.3	-40	98	23.1	-40
**2**	97	24.8	-40	111	26.2	-50
**3**	102	22	-44	97	22.7	-36
**7**	116	22.7	-40	100	23.4	-43
**14**	114	27.2	-43	154	26.9	-40
**30**	102	22.3	-45	135	25.2	-45

Supplementary Table 1: **Stability studies of NanoSTING**

Oligonucleotides	Sequence
Gapdh forward primer	CTCCCACTCTTCCACCTTCG
Gapdh reverse primer	GCCTCTCTTGCTCAGTGTCC
Il6 forward primer	CTGATGCTGGTGACAACCAC
Il6 reverse primer	CAGAATTGCCATTGCACAAC
Il10 forward primer	TGAATTCCCTGGGTGAGAAG
Il10 reverse primer	TTCATGGCCTTGTAGACACC
Ifnb1 forward primer	CTTTGCCATCCAAGAGATGC
Ifnb1 reverse primer	TCTCCCACGTCAATCTTTCC
Ccl5 forward primer	TCGTGTTTGTCACTCGAAGG
Ccl5 reverse primer	AGCAAGCAATGACAGGGAAG
Isg15 forward primer	AAGCAGCCAGAAGCAGACTC
Isg15 reverse primer	CAGTTCTGACACCGTCATGG
Cxcl10 forward primer	CCACGTGTTGAGATCATTGC
Cxcl10 reverse primer	GAGGCTCTCTGCTGTCCATC
Mx1 forward primer	TGTGCAGGCACTATGAGGAG
Mx1 reverse primer	ACTCTGGTCCCCAATGACAG
Mx2 forward primer	AGAGAGACTGACCGCAGAGC
Mx2 reverse primer	TCCTCACTTGCACTCTGGTG
Irf7 forward primer	ACAGCACAGGGCGTTTTATC
Irf7 reverse primer	GAGCCCAGCATTTTCTCTTG
Tnf forward primer	TATGGCTCAGGGTCCAACTC
Tnf reverse primer	CTCCCTTTGCAGAACTCAGG
Ifit1 forward primer	TGCTGAGATGGACTGTGAGG
Ifit1 reverse primer	TCTGGATTTAACCGGACAGC
Ifnl2 forward primer	GAGAAGGACCTGAGGTGCAG
Ifnl2 reverse primer	GGAGTGAATGTGGCTCAGTG
Ifnl3 forward primer	AGGACATGAGGTGCAGTTCC
Ifnl3 reverse primer	GGAGTGAATGTGGCTCAGTG

Supplementary Table 2: Primers used for qRT-PCR for *Mus musculus* (*BALB/c mice*)

Oligonucleotides	Sequence
actb forward primer	CCAAGGCCAACCGTGAAAAG
actb reverse primer	ATGGCTACGTACATGGCTGG
Isg15 forward primer	TCTATGAGGTCCGGCTGACA
Isg15 reverse primer	GCACTGGGGCTTTAGGTCAT
Cxcl11 forward primer	CCGCCTCATACGGGAAATGT
Cxcl11 reverse primer	AAGACAGAAGGTTGGGCTCG
Irf7 forward primer	ATTTCGGTCGCAGGGATCTG
Irf7 reverse primer	TGCAAGATAAAGCGTCCCGT
Ccl5 forward primer	ACTGCCTCGTGTTCACATCA
Ccl5 reverse primer	TTCGGGTGACAAAAACGACT
Il6 forward primer	CCTGAAAGCACTTGAAGAATTCC
Il6 reverse primer	GGTATGCTAAGGCACAGCACACT
Il10 forward primer	GAAGGACCAGCTGGACAACA
Il10 reverse primer	TGGCAACCCAAGTAACCCTTA
Ifnb1 forward primer	AGCTGCATTTCTGCTGTGGT
Ifnb1 reverse primer	CTGCTTTGTCTGGCCTCAAG
Mx2 forward primer	ACCTGACCCTGATTGACCTG
Mx2 reverse primer	ACCAGGTTGATGGTCTCCTG
Mx1 forward primer	AGGAGACCATCAACCTGGTG
Mx1 reverse primer	TCAGGCTTGGTCAAGATTCC

Supplementary Table 3: Primers used for qRT-PCR for *Mesocricetus auratus* (Syrian golden hamster)

Antibody/Conjugate	Color	Cat no; Company; Clone	Dilution
CD11c	BV785	117335; Biolegend; N418	1:80
CD11b	BV421	101235; Biolegend; M1/70	1:100
EPCAM	PerCP-Cy5.5	118219; Biolegend; G8.8	1:100
CD45	APC-Cy7	5642279; BD;30-F11	1:80
CD24	BV605	101817; Biolegend; M1/69	1:100
CD31	PE-Cy7	25031182; Invitrogen; 390	1:40
GS-IB_4 conjugate	AF488	Invitrogen; I21411	1:33.3

Supplementary Table 4: List of antibodies/conjugates used for flow cytometry

Analyte	50 µg dose		250 µg dose	
	Males	Females	Males	Females
Volume (mL)	7.00 ± 5.69	5.58 ± 5.45	6.92 ± 6.57	6.83 ± 0.76
pH	6.83± 1.25	6.08 ± 0.20	6.75 ± 0.88	6.00 ± 0.87
Glucose (mg/dL)	0 ± 0	0 ± 0	0 ± 0	0 ± 0
Ketone (mmol/L)	10.8 ± 6.6	0 ± 0	4.2 ± 5.8	0 ± 0
Protein (mg/dL)	36.7± 31.9	17.5± 14.7	62.5 ± 117.1	0 ± 0
Specific Gravity	1.0200 ± 0.0114	1.0283 ± 0.0041	1.0208 ± 0.0102	1.0233 ± 0.0029
Urobilinogen (EU/dL)	0.2 ± 0.0	0.2 ± 0.0	0.2 ± 0.0	0.2 ± 0.0

Supplementary Table 5: Single dose toxicology study in rats: Urinalysis

Analyte	50 µg dose		250 µg dose	
	Males	Females	Males	Females
APTT (sec)	14.32 ± 0.77	14.2 ± 1.39	12.32 ± 1.84	1.25 ± 0.6
PT (sec)	9.58 ± 0.19	9.27 ± 0.14	9.12 ± 0.35	9.1 ± 0.26

Supplementary Table 6: Single dose toxicology study in rats-Coagulation

Analyte	50 µg dose		250 µg dose	
	Males	Females	Males	Females
ALT (U/L)	31.2 ± 6.6	24.3 ± 7.2	41.2 ± 13.9	24.7 ± 4.2
ALB (g/dL)	4.00 ± 0.15	4.40 ± 0.41	4.07 ± 0.05	4.63 ± 0.4
ALKP (U/L)	199.8 ± 41	79.7 ± 18	289.2 ± 71.5	159 ± 22.6
AST (U/L)	89.8 ± 22.6	84.5 ± 21.3	119 ± 36.5	89 ± 6.2
CALC (mg/dL)	11.15 ± 0.52	10.72 ± 0.84	11.75 ± 0.27	11.57 ± 0.21
CL (mmol/L)	102.33 ± 1.90	100.18 ± 3.15	104.57 ± 0.92	104.23 ± 2.64
CHOL (mg/dL)	68.5 ± 17.4	60.7± 7.6	97.7 ± 18.6	87.7 ± 6.1
CREA (mg/dL)	0.192 ± 0.026	0.235 ± 0.029	0.17 ± 0.00	0.213 ± 0.05
GLOB (g/dL)	2.13 ± 0.18	2.07 ± 0.23	1.2 ± 0.11	1.2 ± 0.35
GLUC (mg/dL)	105.3 ± 31.3	98.8 ± 12.4	65.2 ± 9.2	91.7 ± 16.5
HDL (mmol/L)	1.1 ± 0.297	1.19 ± 0.132	1.7 ± 0.316	1.66 ± 0.108
IPHS (mg/dL)	11.02 ± 1.24	9.68 ± 1.29	13.43 ± 1.05	11.2 ± 1.18
LDL (mmol/L)	0.35 ± 0.084	0.157 ± 0.048	0.483 ± 1.47	0.28 ± 0.082
K (mmol/L)	6.737 ± 1.152	7.572 ± 1.586	8.778 ± 1.618	8.18 ± 2.34
Na (mmol/L)	141.83 ± 2.14	135.83 ± 3.82	139.67 ± 1.21	137 ± 2.65
SDH (U/L)	14.65 ± 4.88	10.42 ± 1.75	37.17 ± 33.92	12.53 ± 3.76
BILI (mg/dL)	0.062 ± 0.01	0.082 ± 0.021	0.087 ± 0.029	0.053 ± 0.015
TP (g/dL)	6.13 ± 0.12	6.47 ± 0.54	5.27 ± 0.08	5.83 ± 0.74
TRIG (mg/dL)	59.2 ± 36.2	33.7 ± 7.9	127.2 ± 50.8	61.7 ± 15.5
BUN (mg/dL)	10.7 ± 1.6	13 ± 1.7	8.7 ± 3.2	12.3 ± 1.5

Supplementary Table 7: Single dose toxicology study in rats-Clinical chemistry

Analyte	50 µg dose		250 µg dose	
	Males	Females	Males	Females
ABAS (x 10^3/µL)	0.06 ± 0.027	0.043 ± 0.012	0.043 ± 0.014	0.04 ± 0.026
AEOS (x 10^3/µL)	0.06 ± 0.028	0.0723 ± 0.017	0.057 ± 0.029	0.073 ± 0.025
ALUC (x 10^3/µL)	0.078 ± 0.055	0.057 ± 0.014	0.107 ± 0.049	0.120 ± 0.036
ALYM (x 10^3/µL)	6.27 ± 0.971	5.108 ± 0.656	5.338 ± 0.843	7.520 ± 0.457
AMON (x 10^3/µL)	0.322 ± 0.145	0.282 ± 0.113	0.382 ± 0.102	0.543 ± 0.110
ANEU (x 10^3/µL)	1.307 ± 0.361	1.107 ± 0.316	1.245 ± 0.354	1.260 ± 0.191
ARET (x 10^3/µL)	328.9 ± 87.308	262.283 ± 37.78	489.017 ± 80.77	288.80 ± 82.568
HCT (%)	51.88 ± 2.87	49.25 ± 3.02	48.37 ± 1.19	47.43 ± 0.67
HGB (g/dL)	16.35 ± 0.83	16.10 ± 0.86	14.40 ± 0.39	14.83 ± 0.38
MCV (fL)	63.45 ± 4.62	57.32 ± 1.56	65.75 ± 1.25	62.00 ± 1.51
MCH (pg)	19.97 ± 1.10	18.70 ± 0.66	19.58 ± 0.39	19.40 ± 0.70
MCHC (g/dL)	31.52 ± 0.56	32.62 ± 0.53	29.97 ± 0.73	31.30 ± 0.35
PLT (x 10^3/µL)	1215.5 ± 177.5	1202.5 ± 180.77	1852.67 ± 189.59	1872.67 ± 140.69
RBC (x 10^6/µL)	8.207 ± 13.45	8.617 ± 0.526	7.357 ± 0.258	7.653 ± 0.103
RDW (%)	13.45 ± 0.89	12.10 ± 0.58	13.92 ± 0.83	11.93 ± 0.76
WBC (x 10^3/µL)	8.089 ± 1.232	6.578 ± 0.670	7.178 ± 1.149	9.560 ± 0.789

Supplementary Table 8: Single dose toxicology study in rats-Hematology

Analyte	Control		125 µg dose (4 doses)	
	Males	Females	Males	Females
Volume (mL)	8.83 **±** 5.8	2.5 ± 1.5	3.83 ± 2.42	3.75 ± 2.62
pH	7.33 ± 1.26	6.33 ± 0.29	6.17 ± 0.61	6.08 ± 0.38
Glucose (mg/dL)	0 ± 0	0 ± 0	16.7 ± 40.8	0 ± 0
Ketone (mmol/L)	11.7 ± 5.8	0 ± 0	10 ± 5.5	0.8 ± 5.5
Protein (mg/dL)	25 ± 8.7	53.3 ± 40.4	107.5 ± 101.8	43.3 ± 101.8
Specific Gravity	1.02 ± 0.0087	1.03 ± 0	1.0292 ± 0.002	1.0283 ± 0.002
Urobilinogen (EU/dL)	0.2 ± 0	0.2 ± 0	0.33 ± 0.33	0.2 ± 0.33

Supplementary Table 9: Repeat dose toxicology study in rats-Urinalysis

Analyte	Control		125 µg dose (4 doses)	
	Males	Females	Males	Females
APTT (sec)	15.5 ± 1.25	14.87 ± 0.51	15.58 ± 0.92	13.93 ± 0.88
PT (sec)	9.9 ± 0.17	9.07 ± 0.12	9.88 ± 0.12	9.30 ± 0.33

Supplementary Table 10: Repeat dose toxicology study in rats-Coagulation

Analyte	Control		125 µg dose (4 doses)	
	Males	Females	Males	Females
ALT (U/L)	27.3 ± 6.5	22.3 ± 3.8	26.8 ± 1.5	22.8 ± 4.5
ALB (g/dL)	3.83 ± 0.06	4.7 ± 0.2	3.88 ± 0.13	4.42 ± 0.39
ALKP (U/L)	151.3 ± 33.1	98 ± 14.7	159.7 ± 22.8	76.5 ± 14.1
AST (U/L)	84 ± 13.7	87.7 ± 26.4	100.2 ± 35.3	96 ± 28.2
CALC (mg/dL)	10.67 ± 0.21	10.83 ± 0.55	10.73 ± 0.5	10.83 ± 0.63
CL (mmol/L)	100.87 ± 0.47	100.77 ± 1.25	101.08 ± 1.71	98.9 ± 1.9
CHOL (mg/dL)	48 ± 17.3	77.7 ± 7.1	63.8 ± 12.4	83 ± 19.3
CREA (mg/dL)	0.19 ± 0.035	0.243 ± 0.04	0.172 ± 0.004	0.213 ± 0.036
GLOB (g/dL)	1.97 ± 0.12	1.87 ± 0.12	1.8 ± 0.19	1.98 ± 0.19
GLUC (mg/dL)	131.7 ± 27.3	142.3 ± 25.7	125.3 ± 17.6	104.5 ± 18.2
HDL (mmol/L)	0.833 ± 0.321	1.67 ± 0.132	1.083 ± 0.214	1.707 ± 0.437
IPHS (mg/dL)	10.27 ± 0.35	8.57 ± 0.42	9.95 ± 0.42	8.85 ± 1.38
LDL (mmol/L)	0.2 ± 0.1	0.147 ± 0.021	0.283 ± 0.075	0.217 ± 0.055
K (mmol/L)	7.043 ± 1.438	5.753 ± 0.074	6.995 ± 0.199	6.582 ± 1.163
Na (mmol/L)	141 ± 0	140.33 ± 1.15	140.67 ± 2.07	138.17 ± 2.48
SDH (U/L)	8.57 ± 7.7	9.37 ± 7.1	9.3 ± 4.64	7.75 ± 4.29
BILI (mg/dL)	0.033 ± 0.006	0.07 ± 0.02	0.037 ± 0.014	0.07 ± 0.03
TP (g/dL)	5.8 ± 0.1	6.57 ± 0.31	5.68 ± 0.26	6.4 ± 0.44
TRIG (mg/dL)	43.7 ± 16.7	36 ± 3.6	54.7 ± 21.5	48.7 ± 8.2
BUN (mg/dL)	15 ± 1.7	14.3 ± 3.2	11.8 ± 1.3	13.2 ± 2

Supplementary Table 11: Repeat dose toxicology study in rats-Clinical chemistry

Analyte	Control		125 µg dose (4 doses)	
	Males	Females	Males	Females
ABAS (x 10^3/µL)	0.06 ± 0.02	0.04 ± 0.017	0.068 ± 0.027	0.043 ± 0.015
AEOS (x 10^3/µL)	0.083 ± 0.031	0.077 ± 0.015	0.105 ± 0.058	0.105 ± 0.032
ALUC (x 10^3/µL)	0.07 ± 0.01	0.08 ± 0.026	0.092 ± 0.019	0.065 ± 0.008
ALYM (x 10^3/µL)	7.523 ± 0.856	5.643 ± 1.235	8.172 ± 1.912	6.162 ± 1.512
AMON (x 10^3/µL)	0.277 ± 0.133	0.247 ± 0.038	0.325 ± 0.132	0.217 ± 0.061
ANEU (x 10^3/µL)	1.917 ± 0.769	1.627 ± 0.375	1.953 ± 0.708	1.3 ± 0.465
ARET (x 10^3/µL)	178.633 ± 15.821	185.267 ± 47.81	157.817 ± 31.355	171.283 ± 31.285
HCT (%)	49.43 ± 2.4	47.8 ± 0.62	50.7 ± 0.88	48.2 ± 1.14
HGB (g/dL)	15.27 ± 0.61	15.03 ± 0.15	15.63 ± 0.23	15.3 ± 0.48
MCV (fL)	62.2 ± 0.17	62.2 ± 1.35	61.93 ± 1.49	60.7 ± 2.31
MCH (pg)	19.23 ± 0.32	19.6 ± 0.53	19.12 ± 0.53	19.3 ± 0.69
MCHC (g/dL)	30.9 ± 0.36	31.47 ± 0.15	30.78 ± 0.49	31.78 ± 0.42
PLT (x 10^3/µL)	1081.33 ± 99.05	1059 ± 53.39	875.17 ± 148.73	1023 ± 84.98
RBC (x 10^6/µL)	7.937 ± 0.421	7.687 ± 0.28	8.178 ± 0.157	7.945 ± 0.356
RDW (%)	11.97 ± 0.31	11.47 ± 0.45	11.83 ± 0.19	11.48 ± 0.73
WBC (x 10^3/µL)	9.927 ± 1.739	7.717 ± 1.518	10.713 ± 2.408	7.895 ± 1.668

Supplementary Table 12: Repeat dose toxicology study in rats: Hematology

Without NanoSTING	With NanoSTING	Nomenclature
$\frac{dT}{dt} = -\beta VT - \varphi IT + \rho R$<br>$\frac{dR}{dt} = \varphi IT - \rho R$<br>$\frac{dE}{dt} = \beta VT - kE$<br>$\frac{dI}{dt} = kE - \sigma I$<br>$\frac{dV}{dt} = \pi I - cV$	$\frac{dT}{dt} = -\beta VT - (\varphi I + \mathrm{NanoSTING})T + \rho R$<br>$\frac{dR}{dt} = (\varphi \mathrm{I} + \mathrm{NanoSTING})\mathrm{T} - \rho R$<br>$\frac{dE}{dt} = \beta VT - kE$<br>$\frac{dI}{dt} = kE - \sigma I$<br>$\frac{dV}{dt} = \pi I - cV$	T = Target cells<br>R = Refractory cells<br>E = Eclipse phase cell (Infected cells not producing virus)<br>I = Infected cells productively making virus<br>V = Viral titer

Supplementary Table 13: Differential equations describing kinetics of SARS-CoV-2 viral infection with and without NanoSTING

Parameter	Description	Mean population value
β	Infectivity parameter constant	$3.2*10^{-8}$mL/RNA copy/day
σ	Death rate of infected cells	1.7 /day
π	Composite parameter for virus production and sampling	45.3/mL/day
ϕ	Rate constant for Interferon induced conversion of Target cells to refractory cells	$1.3*10^{-6}$ /cell/day
k	1/the eclipse phase duration	4 /day
c	virus clearance rate	10 /day
ρ	Rate at which refractory cells become target cells again	0.0044/day

Supplementary Table 14: Mean population parameter values for kinetic model of SARS-CoV-2 viral infection in upper respiratory tract of humans.

Variable	Initial value
T0 – Total number of target cells	$8*10^{7}$
E0 – Initial number of Infected cells	5, 500

Supplementary Table 15: Initial conditions used for solving SARS-CoV-2 kinetic equations for viral infection in upper respiratory tract of humans.

Sup Note 1: MATLAB code

Code for studying the kinetics of viral evolution upon administration of NanoSTING

```
% covid dynamics with NanoSTING
%beta - Infectivity parameter constant = 3.2*10^-8 mL/RNA copy/day
%delta - Death rate of infected cells - 1.7/day
%pii - Composite parameter for virus production and sampling - 45.3/mL/day
%phi - Rate constant for interferon induced conversion of Target cells to
%refractory cells - 1.3*10^-6 /cell/day
%rho - Rate at which refractory cells become target cells again -0.0044/day
%c - Virus clearance rate - 10/day
%k - 1/the eclipse phase duration = 4/day

beta  = 3.2*10^-8; %mL/RNA copy/day
delta = 1.7; %/day
pii = 45.3; %/mL/day
phi = 1.3*10^-6; %/cell/day
rho = 0.0044; %/day
c   = 10; %/day
k   = 4; %/day
nsF = 0; % coefficient relating interferon through NanoSTING

T0 = 8*10^7; %Total number of target cells
R0 = 0; %Initial refractory cells
E0 = 5; %Initial number of infected cells
I0 = 0;
V0 = 0; %Initial virus titer

t_int = [0,30];
init_cond = [T0,R0,E0,I0,V0]';
[t,y] = ode45(@(t,Y) covidode(t,Y,beta,delta,pii,phi,rho,c,k,nsF), t_int,init_cond );
```

Function handle:

```
function dYdt = covidode(t,Y,beta,delta,pii,phi,rho,c,k,nsF )
dYdt = [ -beta*Y(5)*Y(1)-(phi*Y(4)+rectangularPulse(0,1,t)*nsF)*Y(1)+rho*Y(2);
        (phi*Y(4)+rectangularPulse(0,1,t)*nsF)*Y(1)-rho*Y(2);
        beta*Y(5)*Y(1)-k*Y(3);
        k*Y(3)-delta*Y(4);
        pii*Y(4)-c*Y(5)];
end
```
