# Supplementary material for: An intranasal nanoparticle STING agonist protects against respiratory viruses in animal models
Source: Nat Commun. 2024 Jul 18;15:6053. doi: 10.1038/s41467-024-50234-y (PMC11258242; doi:10.1038/s41467-024-50234-y)
Supplement: Supplementary file 3 — Reporting Summary [file 41467_2024_50234_MOESM3_ESM.pdf]

nature portfolio

Corresponding author(s): Navin Varadarajan

Last updated by author(s): Jun 17, 2024

Reporting Summary

Nature Portfolio wishes to improve the reproducibility of the work that we publish. This form provides structure for consistency and transparency in reporting. For further information on Nature Portfolio policies, see our Editorial Policies and the Editorial Policy Checklist.

Statistics

For all statistical analyses, confirm that the following items are present in the figure legend, table legend, main text, or Methods section.

n/a	Confirmed	
☐	☒	The exact sample size (*n*) for each experimental group/condition, given as a discrete number and unit of measurement
☐	☒	A statement on whether measurements were taken from distinct samples or whether the same sample was measured repeatedly
☐	☒	The statistical test(s) used AND whether they are one- or two-sided<br>*Only common tests should be described solely by name; describe more complex techniques in the Methods section.*
☒	☐	A description of all covariates tested
☒	☐	A description of any assumptions or corrections, such as tests of normality and adjustment for multiple comparisons
☐	☒	A full description of the statistical parameters including central tendency (e.g. means) or other basic estimates (e.g. regression coefficient) AND variation (e.g. standard deviation) or associated estimates of uncertainty (e.g. confidence intervals)
☐	☒	For null hypothesis testing, the test statistic (e.g. *F*, *t*, *r*) with confidence intervals, effect sizes, degrees of freedom and *P* value noted<br>*Give P values as exact values whenever suitable.*
☒	☐	For Bayesian analysis, information on the choice of priors and Markov chain Monte Carlo settings
☒	☐	For hierarchical and complex designs, identification of the appropriate level for tests and full reporting of outcomes
☒	☐	Estimates of effect sizes (e.g. Cohen's *d*, Pearson's *r*), indicating how they were calculated

Our web collection on statistics for biologists contains articles on many of the points above.

Software and code

Policy information about availability of computer code

Data collection	Characterization of NanoSTING was performed using DLS and zeta sizer on Litesizer 500 (Anton Paar) in automatic mode. THP1-dual assay, Evan's blue assay and ELISA data was collected using Cytation 7, Bio-Tek Instruments, Inc., Gen5 Software (Version 3.10.06). Reverse Transcription qPCR data was collected using an AriaMx Real-time PCR System (Agilent Technologies, Santa Clara, CA). Preparation of RNA library and mRNA sequencing was conducted by Novogene Co., LTD (Beijing, China).
Data analysis	The statistical analyses were performed in the Prism (GraphPad) software package (version v6.07). Reverse transcription qPCR data was analyzed using the Agilent Aria software (Version 1.8; Applied Biosystems). For analysis of RNA-sequencing data, we paired and trimmed the fastq files using Trimmomatic (v 0.39) and aligned them to the Syrian golden hamster genome (MesAur 1.0, ensembl) using STAR (v 2.7.9a). We determined the differential gene expression using DESeq2 (v 1.28.1) package (PMID: 25516281). To perform gene set enrichment analysis, we used a pre-ranked gene list of differentially expressed genes in GSEA software (UC San Diego and Broad Institute). To generate the gene set for IFN-independent activities of STING, we collected genes with a 2-fold change increase in BMDM-STING S365A-DMXAA vs BMDM-STING S365A-DMSO samples from the GSE149744 dataset as described previously (PMID: 32640258). For quantitative modellng, we solved the system of ODEs for different efficacies, treatment initiation time, and duration of response of NanoSTING using the ODE45 function in MATLAB 2018b. A sample MATLAB code for solving the system of equations has been provided in supplementary methods.

For manuscripts utilizing custom algorithms or software that are central to the research but not yet described in published literature, software must be made available to editors and reviewers. We strongly encourage code deposition in a community repository (e.g. GitHub). See the Nature Portfolio guidelines for submitting code & software for further information.

Data

Policy information about availability of data

All manuscripts must include a data availability statement. This statement should provide the following information, where applicable:

- Accession codes, unique identifiers, or web links for publicly available datasets
- A description of any restrictions on data availability
- For clinical datasets or third party data, please ensure that the statement adheres to our policy

All data are included in the Supplementary Information or available from the authors, as are unique reagents used in this Article. The raw numbers for charts and graphs are available in the Source Data file whenever possible. Sequencing data reported in this paper has been deposited to GEO (GSE201423) and is publically available. All requests for material and experimental data should be directed to the corresponding author, Navin Varadarajan. Source data are provided with this paper.

Research involving human participants, their data, or biological material

Policy information about studies with human participants or human data. See also policy information about sex, gender (identity/presentation), and sexual orientation and race, ethnicity and racism.

Reporting on sex and gender	*Use the terms sex (biological attribute) and gender (shaped by social and cultural circumstances) carefully in order to avoid confusing both terms. Indicate if findings apply to only one sex or gender; describe whether sex and gender were considered in study design; whether sex and/or gender was determined based on self-reporting or assigned and methods used.*<br>*Provide in the source data disaggregated sex and gender data, where this information has been collected, and if consent has been obtained for sharing of individual-level data; provide overall numbers in this Reporting Summary. Please state if this information has not been collected.*<br>*Report sex- and gender-based analyses where performed, justify reasons for lack of sex- and gender-based analysis.*
Reporting on race, ethnicity, or other socially relevant groupings	*Please specify the socially constructed or socially relevant categorization variable(s) used in your manuscript and explain why they were used. Please note that such variables should not be used as proxies for other socially constructed/relevant variables (for example, race or ethnicity should not be used as a proxy for socioeconomic status).*<br>*Provide clear definitions of the relevant terms used, how they were provided (by the participants/respondents, the researchers, or third parties), and the method(s) used to classify people into the different categories (e.g. self-report, census or administrative data, social media data, etc.)*<br>*Please provide details about how you controlled for confounding variables in your analyses.*
Population characteristics	*Describe the covariate-relevant population characteristics of the human research participants (e.g. age, genotypic information, past and current diagnosis and treatment categories). If you filled out the behavioural & social sciences study design questions and have nothing to add here, write "See above."*
Recruitment	*Describe how participants were recruited. Outline any potential self-selection bias or other biases that may be present and how these are likely to impact results.*
Ethics oversight	*Identify the organization(s) that approved the study protocol.*

Note that full information on the approval of the study protocol must also be provided in the manuscript.

Field-specific reporting

Please select the one below that is the best fit for your research. If you are not sure, read the appropriate sections before making your selection.

- [x] Life sciences
- [ ] Behavioural & social sciences
- [ ] Ecological, evolutionary & environmental sciences

For a reference copy of the document with all sections, see nature.com/documents/nr-reporting-summary-flat.pdf

Life sciences study design

All studies must disclose on these points even when the disclosure is negative.

Sample size	No statistical methods were used to predetermine sample sizes for the in-vitro and animal studies.Sample size was determined based on similar studies in this field.
Data exclusions	No data was excluded from the analyses.
Replication	Animal studies were performed in biological triplicates or more as indicated in the figure legends. When applicable, technical repeats is specified for each experiment in the figure legends. Reproducibility between animals in NanoSTING and naïve controls/placebo treated groups is shown in the results.
Randomization	Animals were randomly divided into experimental groups.

Blinding	The researchers were not blinded to allocation during experiments and outcome assessment. Data collection and analysis were not performed blind to the conditions of the experiments. The pathologists performing the histopathological analysis were blinded to treatment. The formulation was manufactured at UH and shipped to USU. All animal experiments at USU were performed independently.

Reporting for specific materials, systems and methods

We require information from authors about some types of materials, experimental systems and methods used in many studies. Here, indicate whether each material, system or method listed is relevant to your study. If you are not sure if a list item applies to your research, read the appropriate section before selecting a response.

Materials & experimental systems

n/a	Involved in the study	
☐	☒	Antibodies
☐	☒	Eukaryotic cell lines
☒	☐	Palaeontology and archaeology
☐	☒	Animals and other organisms
☒	☐	Clinical data
☒	☐	Dual use research of concern
☒	☐	Plants

Methods

n/a	Involved in the study	
☒	☐	ChIP-seq
☐	☒	Flow cytometry
☒	☐	MRI-based neuroimaging

Antibodies

Antibodies used	1.CD11c (BV785, Biolegend #117335; Clone:N418)<br>2. CD11b (BV421, Biolegend #101235; Clone: M1/70)<br>3. EPCAM (PerCP-Cy5.5, Biolegend #118219; Biolegend; G8.8)<br>4. CD45 (APC-Cy7, BD #561037; Clone: 30-F11)<br>5. CD24 (BV605, Biolegend #101817; Clone: M1/69)<br>6. CD31 (PE-Cy7, Invitrogen #25031182; Clone: 390)
Validation	All antibodies are commercially available and validated by manufacturers. Additionally information can be found on product website, listed below.<br>1. https://www.biolegend.com/en-gb/products/brilliant-violet-785-anti-mouse-cd11c-antibody-7963?GroupID=BLG11937<br>2. https://www.biolegend.com/en-ie/products/brilliant-violet-421-anti-mouse-human-cd11b-antibody-7163<br>3. https://www.biolegend.com/en-gb/products/percp-cyanine5-5-anti-mouse-cd326-ep-cam-antibody-5602?GroupID=BLG6455<br>4. https://www.bdbiosciences.com/en-us/products/reagents/flow-cytometry-reagents/research-reagents/single-color-antibodies-ruo/apc-cy-7-rat-anti-mouse-cd45.561037<br>5. https://www.biolegend.com/en-gb/products/alexa-fluor-647-anti-mouse-cd24-antibody-3207?GroupID=BLG4753<br>6. https://www.thermofisher.com/antibody/product/CD31-PECAM-1-Antibody-clone-390-Monoclonal/25-0311-82

Eukaryotic cell lines

Policy information about cell lines and Sex and Gender in Research

Cell line source(s)	NF-κB-SEAP IRF-Luc Reporter Monocytes- THP1-Dual™ Cells (human, Invivogen: Cat No. thpd-nfis)<br>African green monkey kidney cells-VeroE6 (ATCC®, cat# CRL-1586'M)<br>Madin-Darby canine kidney- MDCK cells (ATCC®, cat# CCL-34)
Authentication	All cell lines were purchased directly from Invitrogen and ATCC.
Mycoplasma contamination	The cell lines were tested negative for mycoplasma.
Commonly misidentified lines (See ICLAC register)	No commonly misidentified cell lines were used in this study.

Animals and other research organisms

Policy information about studies involving animals; ARRIVE guidelines recommended for reporting animal research, and Sex and Gender in Research

Laboratory animals	In this study were used Balb/c mice (9-10 weeks old) and Syrian Golden hamsters (Mesocricetus auratus) median age 6 to 10 weeks old. We purchased the animals from Jackson Laboratory and Charles River. Rhesus macaques (Macaca mulatta; Indian origin, between 4 and 11 years of age and 4 – 12 kg in weight) were used.

Wild animals	No wild animals were used in this study.
Reporting on sex	For all the viral challenge studies done at UH and USU, equal number of male and female mice and hamsters were used for the study. For Evan's blue assay, equal number of males and female hamsters were used.
Field-collected samples	No field-collected samples were used in this study.
Ethics oversight	The mouse, hamster and NHP studies were performed under the study protocol (PROTO2020000019, PROTO202100006, PROTO202100049, PROTO202200025), as approved by the Institutional Animal Care and Use Committee in University of Houston. The animal experiments at USU were conducted in accordance with an approved protocol by the Institutional Animal Care and Use Committee of Utah State University. The work was performed in the AAALAC-accredited LARC of the university in accordance with the National Institutes of Health Guide for the Care and Use of Laboratory Animals (8th edition; 2011).

Note that full information on the approval of the study protocol must also be provided in the manuscript.

Plants

Seed stocks	*Report on the source of all seed stocks or other plant material used. If applicable, state the seed stock centre and catalogue number. If plant specimens were collected from the field, describe the collection location, date and sampling procedures.*
Novel plant genotypes	*Describe the methods by which all novel plant genotypes were produced. This includes those generated by transgenic approaches, gene editing, chemical/radiation-based mutagenesis and hybridization. For transgenic lines, describe the transformation method, the number of independent lines analyzed and the generation upon which experiments were performed. For gene-edited lines, describe the editor used, the endogenous sequence targeted for editing, the targeting guide RNA sequence (if applicable) and how the editor was applied.*
Authentication	*Describe any authentication procedures for each seed stock used or novel genotype generated. Describe any experiments used to assess the effect of a mutation and, where applicable, how potential secondary effects (e.g. second site T-DNA insertions, mosiacism, off-target gene editing) were examined.*

Flow Cytometry

Plots

Confirm that:

- [x] The axis labels state the marker and fluorochrome used (e.g. CD4-FITC).
- [ ] The axis scales are clearly visible. Include numbers along axes only for bottom left plot of group (a 'group' is an analysis of identical markers).
- [x] All plots are contour plots with outliers or pseudocolor plots.
- [x] A numerical value for number of cells or percentage (with statistics) is provided.

Methodology

Sample preparation	To isolate lung cells, we perfused the lung vasculature with 5 ml of 1 mM EDTA in PBS without Ca2+, Mg2+ and injected it into the right cardiac ventricle. Nasal tissue and lung was cut into 100–300 mm2 pieces using a scalpel. We transferred the minced tissue to a tube containing 5 ml of digestion buffer containing collagenase D (2mg/ml, Roche #11088858001) and DNase (0.125 mg/ml, Sigma #DN25) in 5 ml of RPMI for 1 h and 30 min at 37 °C in the water bath by vortexing after every 10 min. We disrupted the remaining intact tissue by passage (6-8 times) through a 21-gauge needle. Next, we added 500 µL of ice cold-stopping Buffer (1x PBS, 0.1M EDTA) to stop the reaction. We then removed tissue fragments and dead cells with a 40 µm disposable cell strainer (Falcon) and collected the cells after centrifugation at 400 xg. We then lysed the red blood cells (RBCs) by resuspending the cell pellet in 3 ml of ACK Lysing Buffer (Invitrogen) and incubated for 3 min at RT, followed by centrifugation at 400 xg. Then, we discarded the supernatants and resuspended the cell pellets in 5 ml of complete RPMI medium (Corning, NY, USA). We counted the lung and nasal tissue cells using the trypan blue exclusion method.
Instrument	LSR-Fortessa flow cytometer (BD Bioscience)
Software	FlowJo™ software version 10.8 (Tree Star Inc, Ashland, OR, USA)
Cell population abundance	N/A
Gating strategy	As shown in Supplementary Figure 5, FSC-A vs FSC-H and SSC-H vs SSC-W parameters were used to exclude doublets. In addition, the live cell population was selected as Live/Dead Aqua negative cells. Subsequently, we gated on DiD+SRB+ lung and nasal tissue cells. In both tissues we specifically focused on four major subtypes of cells: epithelial cells (CD45-EPCAM +CD31-), endothelial cells (CD45-CD31+) and two myeloid subsets: CD45+EPCAM-CD11b+CD11c- cells and CD45+EPCAM-CD11c+CD11b- cells. We investigated the cell types of the murine tracheal epithelium (basal cells, secretory cells & ciliated cells). SSC-A: side scatter area, FSC-A: forward scatter area, FSC-H: forward scatter height, SSC-H: side scatter height, SSC-W: side scatter width.

- [x] Tick this box to confirm that a figure exemplifying the gating strategy is provided in the Supplementary Information.
